# Reducing Emissions from Combustion of Grape Residues in Mixtures with Herbaceous Biomass

**DOI:** 10.3390/ma15207288

**Published:** 2022-10-18

**Authors:** Jan Malaťák, Jan Velebil, Jitka Malaťáková, Luboš Passian, Jiří Bradna, Barbora Tamelová, Arkadiusz Gendek, Monika Aniszewska

**Affiliations:** 1 Department of Technological Equipment of Buildings, Faculty of Engineering, Czech University of Life Sciences Prague, Kamýcká 129, 165 00 Prague, Czech Republic; 2 Department of Economics, Faculty of Economics and Management, Czech University of Life Sciences Prague, Kamýcká 129, 165 21 Prague, Czech Republic; 3 Department of Biosystems Engineering, Institute of Mechanical Engineering, Warsaw University of Life Sciences—SGGW, Nowoursynowska 164, 02-787 Warsaw, Poland

**Keywords:** grape pomace, stems, *Miscanthus sinensis*, carbon monoxide, nitrogen oxides, combustion test, excess air co-efficient

## Abstract

The use of grape residues as a renewable energy source for combustion presents various problems. One of these is the excessive production of carbon monoxide and nitrogen oxides. Analyses and combustion tests were performed on white and red grape pomace as well as grape stems. To verify the possibility of a reduction in emissions, straw of *Miscanthus sinensis* was added to mixtures with red grape pomace. Emission concentrations of carbon monoxide and nitrogen oxides were determined on a grate combustion device with a nominal thermal output of 8 kW under steady-state conditions. In addition to these emission concentrations, the excess air factor and the flue gas temperature were monitored. The results show a high energy content in grape residues. In red grape pomace, the gross calorific value of dry matter reached 22.17 MJ kg^−1^. Unfavourable properties included high ash and nitrogen contents. During combustion tests on all types of grape residue, the emission concentrations of carbon monoxide were above the legal limit for the combustion of solid fuels. The addition of Miscanthus straw improved the behaviour during combustion. The maximum content of grape pomace in the mixture capable of meeting legislative emission requirements was 50% wt.

## 1. Introduction

There is a continuous pressure on the increased dependence on renewable energy sources in energy production, including wastes. This use of solid waste biomass is also expected to reduce the production of greenhouse gases [1]. However, some wastes for which there is no economic use still often end up in landfill. One of these are the wastes from the winemaking industry. One way to use them could be as an energy source for combustion. However, the use of raw materials which, which by their nature, are not suitable for direct incineration may lead to some negative effects.

Grapevine is one of the most widespread crops, with world production of more than 50 million tonnes of grapes per year, of which more than 20 million tonnes are accounted for by European producers [2]. Approximately 75% of grapes are used for the production of natural wine [3]. The European Union generates 70% of the world’s production of natural grape wine [4]. From this production comes a significant number of residues from wine processing (seeds, skins, leaves, and stems) produced by the winemaking industry, which causes an environmental burden [5].

Grape pomace is the most abundant solid by-products from wine production [6]. The pomace consists of grape seeds, skins, and, depending on the processing technology, may also contain stems [7]. These residues are a rich source of polysaccharides and phenolic compounds, both flavonoids and non-flavonoids [8,9]. The pomace usually represents 20–30% by weight of the processed grapes [10]. From this percentage, it follows that the winemaking industry worldwide produces millions of tons of residues, which become an ecological and economic problem [5]. In connection with the evaluation of the use of pomace for energy purposes, Pala et al. [11] investigated hydrothermal carbonization and torrefaction, Botelho et al. [12] and Tamelová et al. [13] torrefied grape pomace, Encinar et al. [14] studied pyrolysis, Lapuerta et al. [15] examined pomace gasification, and Miranda et al. [16] evaluated its combustion in the form of pellets.

During the processing of grape wine, waste stems are generated. The stems represent from 3% to 6% of the total weight of grapes [17]. They consist of tissues high in substances such as lignin, cellulose, and hemicellulose, which have a high carbon content [18]. Due to the large number of stems arising during the processing of grapes, the issue of their disposal must be addressed. This also causes economic and environmental problems [5,19]. In practice, their processing involves composting and incorporation into the soil or various forms of biological treatment. Another way to use this waste from grape processing is to use it for solid combustion as a solid fuel in mixtures with wood biomass [16,20].

One of the ways to improve the fuel properties of residues from grape processing is the production of compressed biofuels. Compressed biofuels from waste solid biomass, i.e., briquettes or pellets, will achieve higher energy density, lower moisture, and better fuel homogenization [21]. As other materials with an agro-industrial origin, grape residues show problems with pelletization as well as with emissions of undesirable by-products during combustion [16]. This is the reason why the addition of forest biomass [22] or herbal biomass [23] with good densification properties seems promising for producing briquettes with the required quality.

The initial and most important step in the investigation into the application of fuels made from waste for energy purposes is the determination of calorific values [24], elemental composition, and stoichiometric combustion analysis [25]. The determination of calorific value is the basis for the determination of the heat released from matter upon combustion [26]. The elemental composition indicates the quality and potential applications of any fuel [1]. For other thermochemical processes, it may be necessary to further determine the contents of cellulose, hemicellulose, lignin as well as the content of lipids, proteins, simple sugars, and starches [27,28] for complete characterization.

At present, small combustion units cannot be operated without automation to achieve high efficiency and low emissions. In order to meet the emission limits set for combustion units by the EU [29], these units are operated with a high co-efficient of excess air [30], which is often above three [31]. For optimal operation, it is recommended to maintain 12% vol. oxygen content in the flue gas [32], which corresponds to excess air co-efficient value of 2.33. Emission production is influenced not only by the type of fuel [33], but also by the cycling of the combustion device [34,35]. One approach to minimizing emissions is to increase system efficiency and reduce the number of burn cycles that include start-up and shut-down periods where the emissions are higher [36]. A discontinuous fuel supply mainly leads to increased concentrations of carbon monoxide and nitrogen oxides compared to a continuous fuel supply [37]. Maximum CO_2_ concentrations are reached at low, excess air co-efficients [36] together with the highest combustion temperature [38]. The concentration of nitrogen oxides depends on the combustion temperatures and the amount of combustion air supplied [39].

The aim of this work was to verify the use of grape residues as a renewable energy source for combustion and to reduce the emission concentrations from the combustion of these grape residues below the required legislative limits under real-life conditions. However, because pure grape processing waste significantly exceeded these limits, selected samples were mixed with the straw of *Miscanthus sinensis* to achieve reduced emissions. During the combustion tests, in addition to the emission concentrations of carbon monoxide and nitrogen oxides, the excess air coefficient and the flue gas temperature were monitored.

## 2. Materials and Methods

### 2.1. Materials

Samples of grape pomace were obtained from a winery near Prague as whole batches immediately after pressing in 2020. The white wine variety was Riesling (*Vitis vinifera* “Welschriesling”) and the red wine was Cabernet Sauvignon (*Vitis vinifera* “Cabernet Sauvignon”), both grown in South Moravia. The stems from the Welschriesling variety were sampled from the same batch of grapes as the pomace. These samples were subjected to several modifications before a series of analyses. A sample was taken from each waste material to determine the original moisture. The materials were then dried using ambient air. 

A batch of the Miscanthus sinensis straw was obtained from a sunny plot of land near Prague at the end of March 2020. This material was also airdried after determining the original moisture. Subsequently, the material was ground using a hammer mill. 

A BrikStar CS 25 briquetting press (BRIKLIS, spol. s r.o., Malšice, Czech Republic) was used to produce briquetted samples. It was operated at the maximum hydraulic system pressure of 250 bar. The briquetting was performed with an approximate throughput of 50 kg h^−1^. The nominal diameter of the briquettes was 50 mm. The machine had been working for 1 h before briquetting the tested materials. A minimum of 80 kg of material was used to produce each batch of briquettes.

### 2.2. Fuel Analysis

The fuel parameters determined were moisture, ash, the contents of the main elements, as well as gross and net calorific values. From each briquetted sample, 10 briquettes were sampled which were crushed and mixed. Approximately half of the combined samples was used to determine moisture by drying at 105 °C until constant weight in a laboratory oven Memmert UF30 (Memmert GmbH + Co. KG, Schwabach, Germany). The rest was used to produce representative analytical samples by grinding to a particle size of less than 1 mm using a Retsch SM100 cutting mill (Retsch GmbH., Haan, Germany).

The moisture and ash content in the analytical samples were determined using an automatic thermogravimetric furnace LECO TGA701 (LECO Corporation, St. Joseph, MI, USA) according to ISO 18134-3:2015 [40] and ISO 18122:2015 [41], respectively. They were dried at 105 °C until constant weight and then incinerated at 550 °C in oxygen atmosphere until constant weight. The contents of the main elements (C, H, N) were determined by combustion analysis at 950 °C in LECO CHN628+S analyser (LECO Corporation, St. Joseph, MI, USA). Oxygen was determined as a difference from 100%. Gross calorific value was found by combustion calorimetry in an isoperibol calorimeter LECO AC600 (LECO Corporation, St. Joseph, MI, USA) by combusting 1 g pellets. The conversions for the formation of sulphuric and nitric acid were not performed. Net calorific value was calculated according to ISO 1928:2020 [42]. For each sample, all analyses were made in at least 3 repetitions. 

The analyses results were converted to moisture content in the briquettes and to dry state of the fuel according to ISO 16993:2016 [43].

### 2.3. Combustion Tests

The combustion tests were performed under stabilized conditions on a combustion device with a manually operable grate and manual fuel supply. The combustion chamber has a cross section of 24 × 24 cm and height of 55 cm. The primary combustion air enters the chamber through the grate, the secondary air enters at two locations placed at one, respectively, two thirds of the combustion chamber height. This type of device is suitable for fuels with a large portion of volatile matter. Similar devices are commonly used for domestic heating. This combustion device was also selected for the ability to control the combustion air supply. The ratio between the primary and secondary air was maintained at 3:1. The total amount of air for the individual combustion tests averaged at 11.456 m^3^ per 1 kg of fuel. Overall, the average air consumption stabilized at 22.8 m^3^ h^−1^.

The nominal heat output of the device was 8 kW with the consumption of standard, plant-based fuel specified by the manufacturer at 2.5 kg h^−1^. During the combustion tests, the mass flow of fuel to the combustion device was maintained to stay at the rated heat output. This was based on the elemental composition and calorific values of individual briquette samples. The mass flow of samples during the tests performed for combustion plants with an output of 8 kW at the average of 80% thermal efficiency was calculated based on net calorific values, i.e., for white grape pomace briquettes at 2.02 kg h^−1^, for red grape pomace briquettes at 1.91 kg h^−1^, briquettes from stems at 1.74 kg h^−1^, and from briquettes of Miscanthus straw at 2.29 kg h^−1^. For the briquetted mixtures, the mass flows were based on the percentage of Miscanthus straw. Combustion tests were performed for 6 h for each fuel. The fuel dosing interval was maintained at 30 min. 

Emission concentrations were measured using a Madur GA-60 flue gas analyser (Madur Polska Sp. Z o.o., Zgierz, Poland) along with a flue gas drier from the same manufacturer. During the measurement, the analyser monitored the ambient temperature, the flue gas temperature, and the concentration of O_2_, CO, and nitrogen oxides (the sum of NO and NO_2_) in the flue gas. The sensor signals were proportional to the volume concentration of the measured components in ppm. The concentrations of dry components of the flue gas were converted to normal gas conditions (temperature 0 °C and pressure 101.325 kPa) and the concentration in mg m^−3^ at a reference oxygen content in the flue gas of 13% vol. The analyser had been calibrated by a specialized company and then autocalibrated before each combustion run.

The measured data were obtained under stable combustion conditions at 15-min intervals and each data point was obtained by averaging the analyser output over one minute. During the combustion process, the supply of primary combustion air was controlled. This was monitored by the concentration of oxygen in the flue gas. The co-efficient of excess air was calculated from the emission concentrations and elemental analyses of the samples using Equation (1):

(1) $$n=1+\left(\frac{{\mathrm{CO}}_{2,max}}{{\mathrm{CO}}_{2}}-1\right)\frac{V_{sp,min}}{L_{min}}$$

where: CO_2__,*max*_—theoretical volumetric concentration of carbon dioxide in dry flue gas (% vol.); CO_2_—volumetric concentration of carbon dioxide in dry flue gases (% vol.); *V_sp,min_*_—_theoretical mass amount of dry flue gas (m^3^ kg^−1^); and *L_min_*—theoretical amount of air for complete combustion (m^3^ kg^−1^).

The results of emission measurements were processed by regression analysis by second degree polynomial functions to express the dependence of carbon dioxide and nitrogen oxides on the coefficient of excess air and flue gas temperature.

The work was divided into two phases of combustion tests. In the first phase, the behavior of pure materials was verified. These were three samples: white grape pomace (*GPW-100*), red grape pomace (*GPR-100*), and stems (*GS-100*). Based on the results of the first measurement phase, a reduction in emission concentrations using 25, 50, and 75% of Miscanthus straw in the dry weight of the mixtures was proposed for the second experimental phase. Pure Miscanthus briquettes were also tested. The measurements were repeated under the same conditions as in the first phase of the measurement. The individual briquette samples were assigned the following designations:

- *GPW-100*: pure white grape pomace
- *GPR-100*: pure red grape pomace
- *GS-100*: pure grape stalks
- *MS-100*: pure Miscanthus sinensis straw
- *MS-GPR-25*: 75% wt. Miscanthus straw and 25% wt. red grape pomace
- *MS-GPR-50*: 50% wt. Miscanthus straw and 50% wt. red grape pomace
- *MS-GPR-75*: 25% wt. Miscanthus straw and 75% wt. red grape pomace

## 3. Results and Discussion

### 3.1. Composition Analysis

The proximal and elemental analysis results are listed in Table 1. A problematic parameter, which reduces the usable energy contained in a fuel, is the amount of water. The water content in the original unprocessed materials was around 55% wt. for the pomaces and 70% wt. for the grape stems [44]. This amount of moisture also makes these materials easily susceptible to microbial degradation. After drying the samples under ambient conditions, the lowest moisture content was achieved by Miscanthus straw, which is positive when forming mixtures from waste compacts. The average water content of dried waste from grapes was 9.6% by weight. The moisture in Table 1 corresponds to briquetted samples for combustion tests. Moisture contents below 12% wt. meet the requirements for class A1 wood briquettes according to ISO 17225-3 [45]. Miranda et al. [16] also achieved a very low moisture content in grape waste, which had a positive effect in thermogravimetric combustion tests. Very similar results for mixtures from waste biomass were determined by Gil et al. [20].

One problem making the samples a demanding fuel was the high ash content, which was as high as 5.85% in dry weight of white grape pomace and 7.89 wt. in grape stems. An even higher amount of ash was determined by Toscano et al. [44], who found grape skins to contain 9% wt. of ash in dry matter. Such a large amount of ash reduces the energy density and can cause operational problems for a combustion device, in extreme cases clogging the combustion chamber. It can also lead to increasing the emissions of fly ash in the flue gas, as shown for rapeseed straw [46] or wheat straw [47].

The calorific values of the samples were very close to the results of Toscano et al. [44], differing by less than 3%. Vaštík et. al. [48] also determined similar results. For combustion, the net calorific value of the fuel is the primary parameter. However, the proportion of non-flammable matter in the fuel affects the combustion temperatures as well [49]. Based on stoichiometric calculations (see Table 2), red grape pomace had the best parameters for energy use compared to the other samples. In comparison to wood biomass, both samples of grape pomace had much higher calorific values than spruce or pine wood [50,51], beech wood [52], or Canadian poplar wood [53].

Both grape pomaces had higher carbon, hydrogen, and nitrogen contents compared to the stems as well as the Miscanthus straw. Similar results were reported by Toscano et al. [44] for grape pomace; for the stems, carbon, hydrogen, and nitrogen were slightly lower. High concentrations of nitrogen in the fuel can lead to increased concentrations of nitrogen oxides in the flue gas [39]. Especially in the case of *GPR-100*, where the nitrogen content is quite high for a fuel at 2.13% wt. compared to wood fuel, where the nitrogen concentration tends to be around 0.2% wt. [30]. The elemental composition of Miscanthus straw corresponded to the results of other authors [54,55]. The sulphur content was fairly low, below 0.15% wt. for all samples, which makes these acceptable to use as non-woody-based biofuels. 

The stoichiometric combustion calculations (see Table 2) showed different air consumptions for each sample. The biggest difference was between *GPR-100* and *GS-100*, where the theoretical air consumption and flue gas production differ by 23% on average. Such large differences will significantly differentiate the behaviour of these materials during combustion. In contrast, carbon dioxide concentration with stoichiometric amount of combustion air would be higher with stems, which was determined at 20.53% vol. Similar results were reported by Tamelová et al. [13], who torrefied residues from the processing of grapes for biochar production. The stoichiometric parameters were further used to evaluate emission concentrations in combustion tests.

### 3.2. Combustion Behaviour of Pure Waste Materials from Grapes

**Figure 1:**
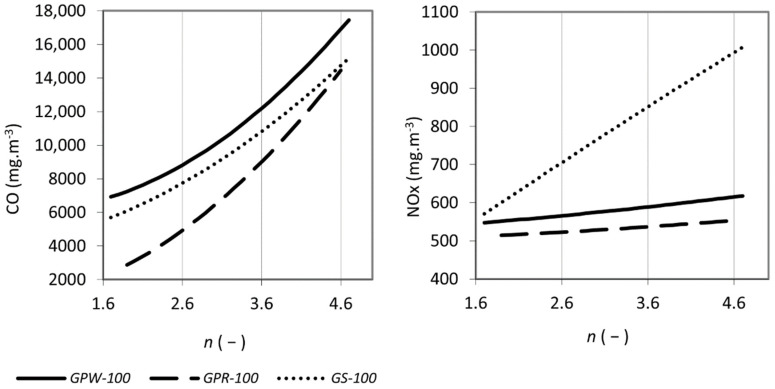
Concentration of CO (**left**) and NOx (**right**) in the flue gas as a function of excess air coefficient *n*, values were converted to reference oxygen content 13% vol. in flue gas and normal gas conditions.

**Figure 2:**
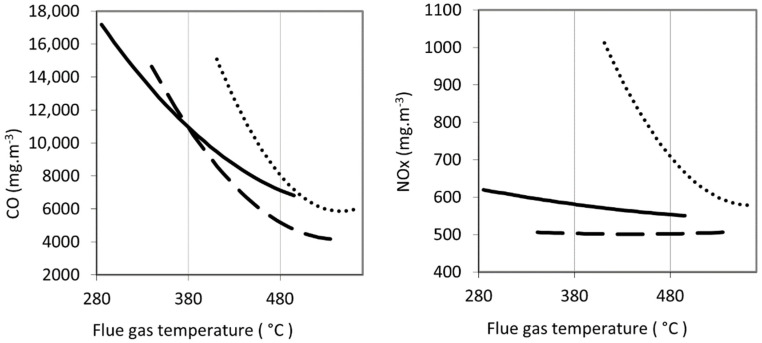
Concentration of CO (**left**) and NOx (**right**) in the flue gas as a function of flue gas temperature, values are converted to reference oxygen content 13% vol. in flue gas and normal gas conditions.

In the first combustion test phase, the aim was to verify the combustion behaviour of pure waste materials from grape processing. The primary results were the emission concentrations of CO and NO_x_ in the flue gas which were expressed as functions of the excess air coefficient and the flue gas temperature in Figure 1 and Figure 2.

During the combustion tests, high average emission concentrations of carbon monoxide were reached by all samples, as evidenced in Table 3. The emission concentrations were high even at their minimum values which were above the legal requirement for this type of combustion plant which is specified at 2000 mg m^−3^ in flue gas converted to reference oxygen content 13% vol. and normal conditions [29]. The requirement for average emission concentrations of nitrogen oxides, where the limit value is 500 mg m^−3^ at the same conditions [29] was not met either. Samples from red grape pomace were the closest to these values. Reducing such high average emission concentrations in combustion plant operation is often difficult [37]. In general, on an existing combustion plant it is possible to reduce emissions by regulating the input of air [56], or by choosing a more suitable fuel [57]. 

**Figure 3:**
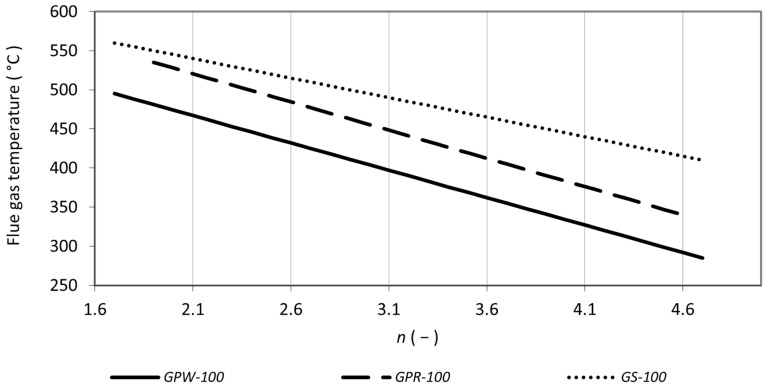
Flue gas temperature as a function of excess air coefficient *n*.

During combustion tests in the first phase, the excess air coefficient was varied in the range between 1.6 and 4.6. Carbon monoxide emission concentration increased with an increasing air excess coefficient for all samples (see Figure 1). The maximum emission concentrations reached was 17,450 mg m^−3^ by *GPW-100*. Such high concentrations have been measured in the combustion of herbal biomass [38] and even exceeded several times in the combustion of agricultural waste [31]. Effective control of the excess air is necessary in such cases and with proper setting the emissions can be reduced below the required limit [36]. Unfortunately, in the present case, even the lowest emission concentrations of carbon monoxide were above 2000 mg m^−3^ and were exceeded several times for white grape pomace and stems. With an increasing excess air ratio, the emission concentrations of carbon monoxide increased in all assessed samples. With increasing temperature, the emission concentrations of carbon monoxide decreased (see Figure 2). This is explained by an increase in the volume of excess air, which decreased the flue gas temperature, (see Figure 3) and thus the carbon monoxide was not completely oxidized [58]. The general trends agree with the results of biomass combustion by Juszczak [37] or Malaťák et al. [25].

The concentration of nitrogen oxides increased with the coefficient of excess air in a similar fashion as shown by Zhou et al. [59]. The nitrogen contained in the combustion air reacts at higher temperatures with oxygen to form NO_x_ [39]. In the case of both grape pomaces, the increase in NO_x_ was gradual with increasing excess air. For *GS-100*, the NO_x_ concentration increased sharply, reaching maximum values above 1000 mg m^−3^. Conversely, with increasing flue gas temperature, NO_x_ concentrations decreased significantly in stems and gradually in pomaces. This does not correspond to the results of Klauser et al. [56] and Winter et al. [35]. This can be explained by the fact that despite the high flue gas temperatures (see Figure 3), a smaller volume of combustion air being involved in the combustion resulted in a decrease in nitrogen oxide production. Similar trends have been shown for herbal biomass [60,61]. Based on the high concentrations of both nitrogen oxides and carbon monoxide, samples of stems were excluded from the second test phase.

### 3.3. Combustion Behaviour of Mixtures of Grape Pomace with Miscanthus Straw

To produce a usable fuel the emission concentrations of carbon monoxide must be reduced below 2000 mg m^−3^ and the emission concentrations of nitrogen oxides below 500 mg m^−3^. The straw of *Miscanthus sinensis* was added into mixtures with the red grape pomace in 25%, 50%, and 75% ratio by dry weight. Red grape pomace was chosen for this measurement because it achieved the lowest emission concentrations out of all grape processing wastes.

The average flue gas emission concentrations shown in Table 4 indicate a positive effect in mixtures of grape pomace with Miscanthus straw. The flue gas temperature has stabilized at around 400 °C, which had an effect on reducing both CO and NO_x_ emission concentrations [62], especially on the production of carbon monoxide emissions [63].

Pure Miscanthus straw had several times lower average emission concentrations of carbon monoxide in the flue gas compared to grape processing wastes. Comparably low emission concentrations were determined during the combustion of waste forest biomass [49]. Lower emission concentrations of nitrogen oxides were also determined, which is directly linked to the flue gas temperature [1].

The mixture with 25% red grape pomace and 75% Miscanthus straw, *MS-GPR-25*, achieved the lowest average emission concentrations of carbon monoxide of all tested samples at any given excess air ratio. The average concentrations of nitrogen oxides were exceeded slightly above the limit of 500 mg m^−3^. A similar positive effect on emission concentrations reduction in mixtures during combustion was reported for separated dried compost [64], as well as in combustion of individual municipal waste components [65].

The mixtures with 50% and 25% red grape pomace indicate the effect of addition of cleanly burning biomass on the emissions of CO and NO_x_ (see Table 4). For mixtures of grape pomace with Pyrenean oak, lower emissions of NO have already been shown by TG–MS measurements [16]. In the present case, adding 25% wt. Miscanthus straw reduced the average carbon monoxide emission concentrations by half compared to pure red grape pomace. Emission concentrations of carbon monoxide were, however, still twice over the limit value. The average nitrogen oxides emission concentrations were above the limit.

**Figure 4:**
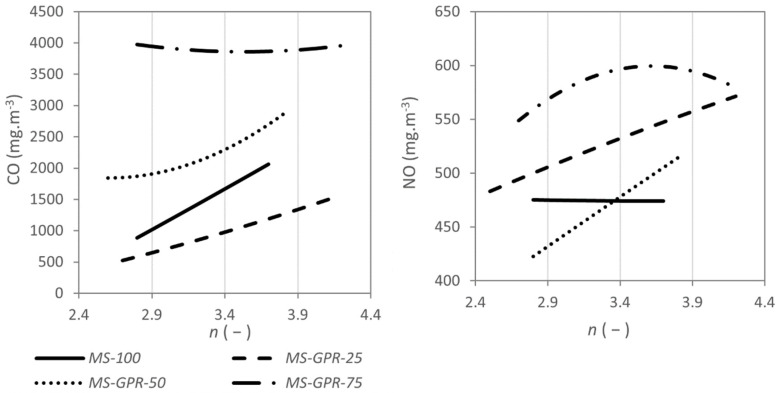
Concentration of CO (**left**) and NOx (**right**) in the flue gas as a function of excess air coefficient *n*, values were converted to reference oxygen content 13% vol. in flue gas and normal gas conditions.

**Figure 5:**
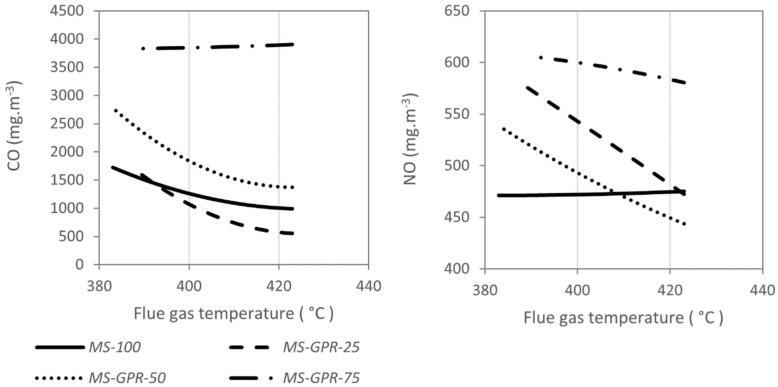
Concentration of CO (**left**) and NOx (**right**) in the flue gas as a function of flue gas temperature, values were converted to reference oxygen content 13% vol. in flue gas and normal conditions.

**Figure 6:**
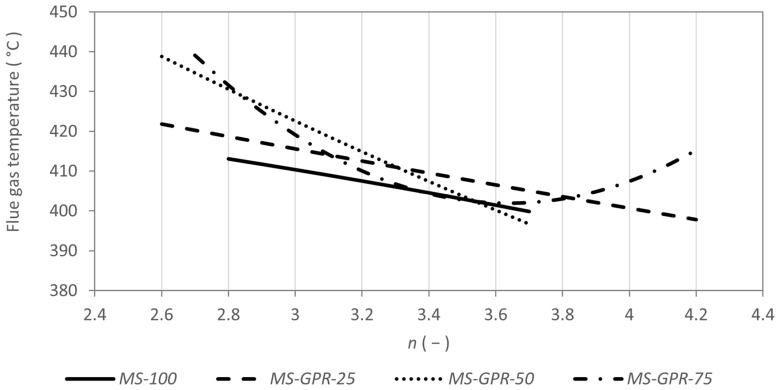
Flue gas temperature as a function of excess air coefficient *n*.

The emission concentrations of carbon monoxide from Miscanthus straw increased with the excess air factor (see Figure 4) as in the combustion of wood fuels [36] and at the same time decreased with increasing flue gas temperature (see Figure 5) as with herbal biomass [57]. Similar trends were also determined for mixtures *MS-GPR-25* and *MS-GPR-50*, where the emission concentrations of nitrogen oxides also increased with excess air. On the other hand, *MS-GPR-75* behaved atypically with the emission concentrations of carbon monoxide being relatively constant between 3831 and 3974 mg m^−3^. Similar emission trends have been reported mainly in the incineration of municipal waste [66] and in some herbal biofuels [67,68]. Such fuels need more combustion air, as can be evident in Figure 6, by the increase in flue gas temperature towards higher excess air ratio. With even more excess air, the emission concentrations of carbon monoxide would increase more significantly [69].

When burning pure Miscanthus straw, NO_x_ emission concentrations stayed nearly constant (see Figure 4 and Figure 5). The same behaviour can be achieved when burning, e.g., pure wood pellets [30,58]. NO_x_ concentrations during the combustion of *MS-GPR-50* and *MS-GPR-25* increased with the coefficient of excess air, which is generally expected in the combustion of waste biomass [34,37,57,63], showing a strong dependence as in [60,61]. The mixture *MS-GPR-75* behaved differently, the NO_x_ emission concentrations increased up to excess air factor of 3.6 where they reached a maximum and then gradually decreased. The flue gas temperature showed the opposite trend having a minimum at excess air factor of 3.6 (see Figure 6). NO_x_ emission concentrations also did not decrease with flue gas temperature as significantly as for other mixtures, however, they were highest of all samples (see Figure 5).

## 4. Conclusions

Three organic wastes generated from wine grape processing (white grape pomace, red grape pomace, and stems from white grapes) were analysed and combusted in briquetted form. The results of compositional analyses confirm some factors making these materials suboptimal fuels, namely the amount of ash and nitrogen. These properties make these wastes problematic for energy use in respect to e.g., the European technical standards for solid biofuels. However, when dried, the calorific is quite high for pomaces, even significantly higher than most types of wood. Stoichiometric combustion calculations showed different consumptions of air as well flue gas volumes for these samples compared to typical fuels. Therefore, the combustion device must be able to supply an appropriate amount of combustion air and regulate its supply while adhering to low values of the coefficient of excess air, which is a necessity for low production of carbon monoxide and nitrogen oxide emissions.

Combustion tests were split into two phases. In the first phase, pure briquetted materials were tested. Emission limits for carbon monoxide concentrations were exceeded by all materials. The emission concentrations of carbon monoxide increased significantly with excess air factor. The lowest values achieved were above 2800 mg m^−3^ for red grape pomace and the maximum reached 17,450 mg m^−3^ for white grape pomace. The nitrogen oxides were also above the 500 mg m^−3^, i.e., over the legal limit. 

In the second phase, red grape pomace was chosen to produce mixtures with straw of *Miscanthus sinensis* to reduce harmful emission concentrations. In all mixtures there was a significant reduction in the emission concentrations of carbon monoxide while maintaining the same rated thermal output compared to pure grape pomace. The highest CO emission concentrations of up to 3974 mg m^−3^ were determined for a mixture with 75% wt. grape pomace. The change in nitrogen oxides emissions was not as favourable as for carbon monoxide. With an increasing proportion of Miscanthus straw, the emission concentrations of nitrogen oxides decreased. As opposed to pure grape pomace, emission concentrations of nitrogen oxides increased significantly with the excess air coefficient. In the case of the mixture with 75% wt. grape pomace, the average concentrations were even higher than in pure pomace. The maximum content of grape pomace in the mixture capable of meeting the legislative emission requirements was 50% wt.

## Figures and Tables

**Table 1:** The results of proximate, elemental, and calorimetric analysis.

	Water Content (% wt.)	Ash (% wt.)	Gross Calorific Value (MJ kg^−1^)	Net Calorific Value (MJ kg^−1^)	Carbon (% wt.)	Hydrogen (% wt.)	Nitrogen (% wt.)	Sulphur (% wt.)	Oxygen (% wt.)
*GPW-100* o.s.	10.80 ±0.50	5.22 ±0.13	19.30 ±0.12	17.84 ±0.20	46.52 ±0.52	5.54 ±0.15	1.47 ±0.11	0.06 ±0.02	30.39 ±0.71
*GPW-100* d.s.	-	5.85 ±0.34	21.65 ±0.18	20.29 ±0.19	52.15 ±0.66	6.21 ±0.17	1.64 ±0.13	0.07 ±0.03	34.08 ±0.77
*GPR-100* o.s.	8.36 ±0.54	4.80 ±0.20	20.30 ±0.14	18.88 ±0.22	48.73 ±0.67	5.58 ±0.19	1.95 ±0.15	0.11 ±0.03	30.47 ±0.81
*GPR-100* d.s.	-	5.24 ±0.22	22.17 ±0.19	20.84 ±0.20	53.18 ±0.79	6.09 ±0.20	2.13 ±0.17	0.12 ±0.03	33.24 ±0.87
*GS-100* o.s.	9.64 ±0.70	7.13 ±0.47	15.80 ±0.11	14.48 ±0.21	42.04 ±0.48	4.96 ±0.16	0.62 ±0.06	0.04 ±0.02	35.57 ±0.76
*GS-100* d.s.	-	7.89 ±0.52	17.49 ±0.16	16.29 ±0.17	46.53 ±0.60	5.49 ±0.17	0.68 ±0.06	0.04 ±0.03	39.37 ±0.81
*MS-100* o.s.	7.81 ±0.53	4.67 ±0.10	17.29 ±0.09	15.94 ±0.17	44.17 ±0.43	5.32 ±0.13	0.94 ±0.03	0.08 ±0.02	37.01 ±0.57
*MS-100* d.s.	-	5.07 ±0.11	18.75 ±0.14	17.50 ±0.15	47.91 ±0.54	5.77 ±0.14	1.02 ±0.03	0.08 ±0.03	40.15 ±0.57

o.s.—original briquette sample, d.s.—dry state, errors signify 95% confidence interval, elemental contents were converted to their value in combustible matter.

**Table 2:** Air consumption and flue gas production from stoichiometric combustion.

Sample	Theoretical Amount of Combustion Air		Theoretical Amount of Dry Flue Gas		Theoretical Concentration of Carbon Dioxide in Dry Flue Gas	
	(kg kg^−1^)	(m^3^ kg^−1^)	(kg kg^−1^)	(m^3^ kg^−1^)	(% wt.)	(% vol.)
*GPW-100*—o.s.	5.95	4.58	6.21	4.45	27.47	19.38
*GPR-100*—o.s.	6.22	4.79	6.49	4.66	27.52	19.40
*GS-100*–o.s.	5.01	3.86	5.33	3.80	28.92	20.53
*MS-100*–o.s.	5.31	4.09	5.65	4.02	28.72	20.37

o.s.—original briquette sample.

**Table 3:** Emission concentrations from combustion of grape residues converted to reference oxygen content 13% vol. in flue gas and normal gas conditions.

	*T_fg_*	*n*	CO	NOx
	(°C)	(−)	(mg m^−3^)	(mg m^−3^)
***GPW-100* original briquette sample**				
Mean	411.61	3.56	10,270.74	572.99
SD	77.64	1.33	3551.40	25.25
Max	495.00	4.70	17,450.00	619.60
Min	285.00	1.71	5410.00	546.40
***GPR-100* original briquette sample**				
Mean	440.73	3.25	7865.37	532.46
SD	60.18	0.61	2845.93	2.13
Max	538.00	4.65	14,450.00	553.48
Min	340.00	1.92	2868.09	514.50
***GS-100* original briquette sample**				
Mean	465.64	3.28	9823.39	730.88
SD	43.80	0.93	3342.19	143.59
Max	560.00	4.71	15,180.35	1020.00
Min	410.00	1.75	5691.51	581.00

SD—standard deviation.

**Table 4:** Emission concentrations from combustion of grape residues in mixtures with Miscanthus straw converted to reference oxygen content 13% vol. in flue gas and normal gas conditions.

	*T_fg_*	*n*	CO	NOx
	(°C)	(−)	(mg m^−3^)	(mg m^−3^)
***MS-100***				
Mean	403	3.2	1365.00	473.51
SD	12.41	0.62	313.51	0.81
Max	423.00	4.20	2060.15	475.28
Min	383.00	2.20	884.34	471.06
***MS-GPR-25***				
Mean	406	3.05	984.48	528.13
SD	10.68	0.53	334.25	30.55
Max	423.00	3.90	1623.69	581.90
Min	389.00	2.20	523.95	472.33
***MS-GPR-50***				
Mean	403	3.20	2023.81	496.43
SD	11.86	0.59	398.03	40.18
Max	423.00	4.20	2861.82	585.67
Min	383.00	2.20	1370.61	422.85
***MS-GPR-75***				
Mean	405	3.10	3880.98	589.85
SD	11.25	0.56	30.42	11.93
Max	423.00	4.00	3974.49	606.17
Min	387.00	2.20	3831.04	548.74

SD—standard deviation.

## Data Availability

The data not directly presented in the article will be made available on request.
